# Influence of neural monitoring during thyroid surgery on nerve integrity and postoperative vocal function

**DOI:** 10.1002/bjs5.50

**Published:** 2018-04-25

**Authors:** A. F. Engelsman, S. Warhurst, S. Fraser, D. Novakovic, S. B. Sidhu

**Affiliations:** ^1^ Endocrine Surgery Unit University of Sydney Sydney New South Wales Australia; ^2^ Voice Research Laboratory, Faculty of Health Sciences University of Sydney Sydney New South Wales Australia

## Abstract

**Background:**

Integrity of the recurrent laryngeal nerve (RLN) and the external branch of the superior laryngeal nerve (EBSLN) can be checked by intraoperative nerve monitoring (IONM) after visualization. The aim of this study was to determine the prevalence and nature of voice dysfunction following thyroid surgery with routine IONM.

**Methods:**

Thyroidectomies were performed with routine division of strap muscles and nerve monitoring to confirm integrity of the RLN and EBSLN following dissection. Patients were assessed for vocal function before surgery and at 1 and 3 months after operation. Assessment included use of the Voice Handicap Index (VHI) 10, maximum phonation time, fundamental frequency, pitch range, harmonic to noise ratio, cepstral peak prominence and smoothed cepstral peak prominence.

**Results:**

A total of 172 nerves at risk were analysed in 102 consecutive patients undergoing elective thyroid surgery. In 23·3 per cent of EBSLNs and 0·6 per cent of RLNs nerve identification required the assistance of IONM in addition to visualization. Nerve integrity was confirmed during surgery for 98·8 per cent of EBSLNs and 98·3 per cent of RLNs. There were no differences between preoperative and postoperative VHI‐10 scores. Acoustic voice assessment showed small changes in maximum phonation time at 1 and 3 months after surgery.

**Conclusion:**

Where there is routine division of strap muscles, thyroidectomy using nerve monitoring confirmation of RLN and EBSLN function following dissection results in no clinically significant voice change.

## Introduction

Thyroidectomy is the most frequently performed endocrine operation in the world. One of the significant complications is vocal fold paralysis from recurrent laryngeal nerve (RLN) injury, although vocal fold paresis resulting from partial loss of nerve function can also cause postoperative dysphonia. The standard to avoid vocal fold paresis is direct visualization, dissection and protection of the RLN^1^. Using this technique, nerve injury rates have been low, with a temporary RLN palsy rate of 3–8 per cent and a permanent rate of 0·3–3 per cent reported^2^. The reported prevalence of external branch of the superior laryngeal nerve (EBSLN) damage is higher. EBSLN damage produces changes in voice that are heterogeneous and difficult to diagnose. A lesion of the EBSLN impairs motor function of the cricothyroid muscle, affecting the production of high tones, limiting pitch range and voice projection, and leading to phonatory fatigue^3^ ^4^. The rate of EBSLN injury has been reported to be as high as 28 per cent^5^.

Intraoperative nerve monitoring (IONM) was developed to reduce the incidence of these problems, and in one RCT^6^ decreased the rate of temporary RLN palsy from 5·0 to 2·7 per cent (*P* = 0·007). Owing to the low RLN injury rate, most studies are, however, underpowered to detect statistically significant differences and, as a result, the routine use of IONM remains controversial. In response to variations in practice and conflicting data, a consensus statement in 2011 by the International Neural Monitoring Study Group^7^ emphasized the importance of standardizing the use of IONM in RLN identification as an adjunct to visualization. When used in a standard fashion during thyroid surgery, nerve monitoring has been shown to improve identification of both the EBSLN and RLN compared with visualization alone^8^. This is especially the case for the EBSLN. An RCT^9^ found significantly less severe impairment of the voice handicap score in monitored patients than in those who had direct visualization alone (2·6 *versus* 11·2 per cent respectively; *P* = 0·021).

In addition, reports suggest that damage to the strap muscles can lead to voice impairment^10^, although no causal relation between voice changes and transection of the strap muscles has been established.

The aim of the present study was to determine the prevalence and nature of voice dysfunction following thyroid surgery with IONM and routine strap muscle division.

## Methods

A cohort of consecutive patients undergoing thyroidectomy who completed informed consent and preoperative and postoperative vocal function assessment were included in the study. Operations were performed by the same single surgeon. Data were held in a specific database approved by the Northern Sydney Area ethics committee. Videostroboscopy and voice assessment were performed on all patients before surgery and at 1 and 3 months after surgery by an independent laryngologist and speech–language therapist. Demographics, type of operation, pathology, visualization of EBSLN and RLN, findings at IONM, EBSLN subtype classification^11^ and complications were recorded. The Voice Handicap Index (VHI) 10 questionnaire result was used as the primary outcome measure. Secondary outcome measurements were based on objective acoustic analysis of recorded voice samples. The preoperative voice outcome assessment functioned as the control for postoperative tests.

### Voice Handicap Index‐10

The VHI‐10 is a validated short version of the 30‐item VHI questionnaire consisting of the ten most robust items^12^. This questionnaire is used to assess the patient's voice‐related quality of life before surgery and 1 and 3 months after surgery.

### Acoustic analysis

Standardized voice recordings were taken in a quiet room with background noise minimized to less than 50 dB and captured with a Quad‐Capture USB sound card (Roland Corporation, Nakagawa, Japan) and an AKG C540 head‐mounted cardioid microphone (Harman International, Stamford, Connecticut, USA), positioned 5 cm from the edge of the lips. The recordings were captured with WavePad audio software 2015 (NCH Software, Greenwood Village, Colorado, USA) at 44 100 Hz, 16‐bit sample rate, and saved as a .wav file. The text and sentences were standardized (*Appendix* S1, supporting information).

Participants were asked to read the Rainbow Passage as a connected speech sample, to produce an /a/ vowel for as long as they could and produce a pitch glide task on /i/. The recordings were analysed for fundamental frequency of connected speech (*f*0), harmonic to noise ratio on a 4‐s segment of prolonged /a/ vowel (HNR), and minimum and maximum frequency (on the pitch glide task) using the Praat speech tool version 5.3.75^13^. The speech tool was used for the cepstral peak prominence (CPP) and smoothed cepstral peak prominence (CPPS) analyses, performed on the connected speech sample^14^. Maximum phonation time (MPT) was taken from the longest /a/ vowel produced in three attempts and was measured in seconds in WavePad once the recording was complete.

### Surgical protocol

Thyroidectomy was performed in a standard fashion under general anaesthesia and cervical block; intubation was with the TriVantage® EMG endotracheal tube (Medtronic, Jacksonville, Florida, USA). The patient was positioned supine with a shoulder roll providing neck extension. A cervical incision in a skin crease was performed and the strap muscles were routinely transected, preserving innervation by the ansa cervicalis, and resuturing with a 3/0 polyglactin suture (Vicryl™; Ethicon, Somerville, New Jersey, USA) at the conclusion of the procedure. The LigaSure™ (Medtronic) thermal sealing system was used for dissection and ligation. IONM was performed with the NIM® 3.0 system (Medtronic). Before dissection of the thyroid, the system was checked by vagal response.

Visual identification of the EBSLN was carried out as reported previously^15^ and confirmed using the nerve monitor. A positive result was recorded when strong and simultaneous contraction of the pars recta and pars oblique components of the cricothyroid muscle was observed. The RLN was also identified and dissected over the course of the nerve, with monitoring again used to confirm nerve function. In all patients with loss of signal, contralateral stimulation of the vagus nerve was performed to exclude equipment failure and palpation for a cricopharyngeal twitch on ipsilateral stimulation of the RLN and vagus nerve. Failure to confirm the integrity of the nerve after dissection was recorded.

### Statistical analysis

Statistical analysis was performed using SPSS® version 24.0 (IBM, Armonk, New York, USA) data analysis software. Baseline characteristics were described as mean(s.d.) or median (range) values for continuous variables, and as counts with percentages for categorical variables. For continuous variables, differences between groups per time point were calculated with ANOVA for independent samples. The Kruskal–Wallis test was used to compare medians between all groups per time point. The significance of change over time per outcome for continuous variables was determined with the generalized linear mixed model for paired data. VHI‐10 outcomes are ordinal and thus *P* values were calculated using a generalized estimating equation model for repeated measures. Outcomes with incomplete data over time were excluded from analysis. *P* < 0·050 was considered significant.

## Results

Of 102 patients, 70 (69 per cent) underwent total thyroidectomy and 32 (31 per cent) had a hemithyroidectomy, resulting in a total of 172 EBSLNs and RLNs at risk (*Tables* 1 and 2). All nerves at risk were identified during surgery, but 23·3 per cent of EBSLNs and 0·6 per cent of RLNs required the assistance of IONM as well as visualization. Nerve integrity was confirmed during surgery for 98·8 per cent of EBSLNs and 98·3 per cent of RLNs. There were no significant differences between median preoperative and postoperative VHI‐10 scores, or in objective outcome measures over the three different time points for all patients combined (*Table* 3).

**Table 1:** Baseline characteristics of patients and nerves at risk

	No. of patients (*n* = 102)
Age (years)*	54·5 (21–84)
Sex ratio (F : M)	85 : 17
Type of surgery	
Total thyroidectomy	70 (68·6)
Hemithyroidectomy	32 (31·4)
Lymph node dissection	15 (14·7)
Indication for surgery	
Risk of malignancy	43 (42·2)
Goitre	43 (42·2)
Thyrotoxicosis	14 (13·7)
Other	2 (2·0)
Histopathology	
Goitre	54 (52·9)
Thyroiditis	4 (3·9)
Other benign	22 (21·6)
Carcinoma	22 (21·6)

Values in parentheses are percentages unless indicated otherwise;

* values are median (range).

**Table 2:** Baseline characteristics of nerves at risk

	No. of nerves at risk		
	Right side (*n* = 82)	Left side (*n* = 90)	Total (*n* = 172)
EBSLN			
Cernea classification^11^			
Type 1	23 (28)	44 (49)	67 (39·0)
Type 2a	42 (51)	27 (30)	69 (40·1)
Type 2b	10 (12)	8 (9)	18 (10·5)
Missing	7 (9)	11 (12)	18 (10·5)
IONM‐assisted identification	20 (24)	20 (22)	40 (23·3)
Loss of signal	1 (1)	1 (1)	2 (1·2)
RLN			
IONM‐assisted identification	0 (0)	1 (1)	1 (0·6)
Not identified	0 (0)	1 (1)	1 (0·6)
Missing	2 (2)	2 (2)	4 (2·3)
Loss of signal	3 (4)	0 (0)	3 (1·7)

Values in parentheses are percentages. EBLSN, external branch of the superior laryngeal nerve; IONM, intraoperative nerve monitoring; RLN, recurrent laryngeal nerve.

**Table 3:** Results of the objective patient‐reported outcome according to the Voice Handicap Index‐10 over time

			Voice Handicap Index‐10 score*			
	Before surgery	*P* †	1 month	*P* †	3 months	*P* †
Total thyroidectomy		0·015		0·464		0·221
No	5·5 (0–29)		3·0 (0–29)		2·0 (0–19)	
Yes	1·0 (0–21)		2·0 (0–34)		1·5 (0–23)	
Lymph node dissection		0·022		0·650		0·515
No	2·0 (0–29)		2·0 (0–34)		2·0 (0–23)	
Yes	0·0 (0–5)		3·0 (0–17)		1·5 (0–11)	
Loss of signal		0·456		0·538		0·888
No	1·0 (0–29)		2·0 (0–29)		2·0 (0–23)	
Yes	2·0 (0–21)		2·0 (0–34)		1·0 (0–11)	
Cancer		0·175		0·805		0·898
No	2·0 (0–29)		2·0 (0–34)		2·0 (0–23)	
Yes	0·5 (0–13)		4·0 (0–17)		2·0 (0–13)	

* Values are median (range).

† Kruskal–Wallis test.

### Voice Handicap Index‐10

Type of thyroidectomy did not influence objective voice outcomes (*Table* 3). The preoperative VHI‐10 score was significantly higher in patients who had a hemithyroidectomy or no lymph node dissection. The VHI‐10 score did not change after 1 and 3 months with respect to the different predictors (*Table* 3). Intraoperative loss of signal in either nerve seemed to be a significant predictor of postoperative change in the VHI‐10 score, although median VHI‐10 changes in patients with and without intraoperative loss of signal showed no clinically relevant differences (*Table* 3).

### Acoustic analysis

Patients had a significantly lower *f*0 (highest) value at 1 month after surgery compared with the preoperative value (461·3 *versus* 515·2 Hz respectively; *P* = 0·005) (*Table* 4). After 3 months there was complete recovery. Overall, all objective voice outcomes showed no changes 3 months after surgery compared with preoperative values (*Table* 4). Patients who underwent total thyroidectomy had significantly lower *f*0 (highest) values before surgery and at 1 month compared with those having a hemithyroidectomy. This difference tended to disappear at 3 months (*Table* 5). There were no differences in acoustic outcomes between patients who underwent lymph node dissection and those who did not (*Table* 6). MPT was significantly reduced 1 month after surgery in patients who had loss of signal during surgery, but it returned to the preoperative baseline after 3 months (*Table* 7). CPP and CPPS were significantly lower after 3 months in patients who had loss of signal during operation (*Table* 7). Histopathological findings were stratified by benign or malignant outcome. Malignant outcome included all cancer subtypes and sizes, with a mean of 22 (range 4–80) mm. Histopathological findings did not influence objective voice outcome measures (*Table* 8).

**Table 4:** Overall changes in objective outcome measurements over time

			After surgery*		
	*n*	Before surgery*	1 month	3 months	*P* ‡
MPT (s)	99	17·1(7·1)	15·2(6·7)	13·4(7·2)	0·854
*f*0 (Hz)	85	190·4(51·6)	194·6(49·8)	194·4(43·4)	0·418
*f*0 (lowest) (Hz)	82	128·9(33·1)	125·8(38·6)	128·3(31·1)	0·782
*f*0 (highest) (Hz)	82	515·2(218·9)	461·3(200·1)	505·5(202·2)	0·654
HNR (Hz)	83	20·4(3·7)	20·3(4·2)	20·5(3·8)	0·774
CPP (dB)	80	12·3(0·8)	12·2(0·8)	12·3(0·8)	0·957
CPPS (dB)	79	4·2(0·5)	4·1(0·5)	4·2(0·5)	0·307
VHI‐10	99	2·0 (0–6)†	2·0 (0–8)†	2·0 (0–6)†	0·326§

* Values are mean(s.d.) unless indicated otherwise;

† values are median (i.q.r.). MPT, maximum phonation time; *f*0, fundamental frequency; HNR, harmonic to noise ratio; CPP, cepstral peak prominence; CPPS, smoothed cepstral peak prominence; VHI, Voice Handicap Index.

‡ Generalized linear mixed model, except

§ generalized estimating equation.

**Table 5:** Objective voice outcome measures for each time point, stratified for type of thyroidectomy (hemithyroidectomy versus total thyroidectomy)

			After surgery			
Total thyroidectomy	Before surgery*	*P* †	1 month*	*P* †	3 months*	*P* †
MPT (s)		0·264		0·228		0·183
No	18·3(7·3)		16·5(6·2)		15·0(6·6)	
Yes	16·6(7·0)		14·8(7·0)		12·9(7·5)	
*f*0 (Hz)		0·274		0·865		0·312
No	177·5(66·1)		196·0(57·0)		187·6(44·6)	
Yes	190·5(49·6)		194·1(48·8)		197·4(41·7)	
*f*0 (lowest) (Hz)		0·759		0·853		0·407
No	128·5(35·7)		125·4(46·4)		124·1(34·2)	
Yes	126·2(33·7)		126·9(33·6)		130·0(30·1)	
*f*0 (highest) (Hz)		0·040		0·026		0·055
No	572·8(250·0)		524·1(225·8)		567·0(213·1)	
Yes	478·2(185·9)		431·7(193·0)		479·7(190·8)	
HNR (Hz)		0·513		0·157		0·314
No	20·6(2·9)		19·5(3·9)		20·0(4·1)	
Yes	20·0(4·7)		20·8(4·1)		20·1(3·8)	
CPP (dB)		0·311		0·797		0·506
No	12·1(0·8)		12·2(0·8)		12·3(0·7)	
Yes	12·3(0·8)		12·2(0·8)		12·2(0·8)	
CPPS (dB)		0·222		0·510		0·722
No	4·1(0·5)		4·1(0·6)		4·2(0·6)	
Yes	4·2(0·6)		4·2(0·6)		4·2(0·6)	

* Values are mean(s.d.). MPT, maximum phonation time; *f*0, fundamental frequency; HNR, harmonic to noise ratio; CPP, cepstral peak prominence; CPPS, smoothed cepstral peak prominence.

† ANOVA.

**Table 6:** Objective voice outcome measures for each time point, stratified for lymph node dissection

			After surgery*			
Lymph node dissection	Before surgery*	*P* †	1 month	*P* †	3 months	*P* †
MPT (s)		0·547		0·212		0·119
No	16·9(6·3)		15·0(6·3)		13·1(6·9)	
Yes	18·2(11·3)		17·4(9·4)		16·4(8·7)	
*f*0 (Hz)		0·836		0·350		0·957
No	186·8(53·6)		192·8(51·9)		194·0(43·2)	
Yes	183·5(67·4)		207·2(47·3)		194·7(41·6)	
*f*0 (lowest) (Hz)		0·333		0·619		0·375
No	128·2(32·3)		125·7(38·3)		126·8(31·6)	
Yes	118·3(45·8)		131·6(37·6)		135·9(31·7)	
*f*0 (highest) (Hz)		0·014		0·990		0·490
No	527·5(211·7)		462·4(201·5)		515·5(212·0)	
Yes	374·0(154·0)		463·1(123·4)		470·2(106·5)	
HNR (Hz)		0·050		0·851		0·637
No	20·5(4·1)		20·3(4·1)		20·6(3·9)	
Yes	18·1(4·1)		20·5(3·9)		20·1(3·1)	
CPP (dB)		0·778		0·514		0·771
No	12·2(0·8)		12·2(0·8)		12·3(0·8)	
Yes	12·3(0·9)		12·1(1·2)		12·3(1·0)	
CPPS (dB)		0·930		0·711		0·987
No	4·2(0·6)		4·2(0·5)		4·2(0·6)	
Yes	4·2(0·6)		4·1(0·6)		4·2(0·6)	

* Values are mean(s.d.). MPT, maximum phonation time; *f*0, fundamental frequency; HNR, harmonic to noise ratio; CPP, cepstral peak prominence; CPPS, smoothed cepstral peak prominence.

† ANOVA.

**Table 7:** Objective voice outcome measures for each time point, stratified for intraoperative loss of signal of any nerve

			After surgery*			
Loss of signal	Before surgery*	*P* †	1 month	*P* †	3 months	*P* †
MPT (s)		0·671		0·006		0·887
No	17·2(7·3)		15·7(6·6)		13·6(7·4)	
Yes	15·8(2·6)		7·2(6·7)		13·1(2·1)	
*f*0 (Hz)		0·894		0·570		0·694
No	186·2(56·5)		194·2(51·8)		194·5(43·6)	
Yes	189·6(27·5)		211·4(33·1)		186·7(25·9)	
*f*0 (lowest) (Hz)		0·440		0·948		0·472
No	126·3(34·6)		126·5(38·7)		128·6(31·3)	
Yes	138·5(26·4)		125·0(5·9)		118·1(37·9)	
*f*0 (highest) (Hz)		0·995		0·253		0·395
No	507·1(215·2)		466·6(194·6)		514·4(204·9)	
Yes	507·7(116·0)		336·7(49·8)		434·7(136·2)	
HNR (Hz)		0·002		0·900		0·563
No	20·5(3·7)		20·3(4·0)		20·5(3·8)	
Yes	14·5(8·7)		20·6(6·2)		21·5(2·1)	
CPP (dB)		0·172		0·110		0·039
No	12·3(0·8)		12·2(0·8)		12·3(0·8)	
Yes	11·8(0·8)		11·4(0·8)		11·6(1·0)	
CPPS (dB)		0·173		0·448		0·039
No	4·2(0·6)		4·2(0·6)		4·2(0·5)	
Yes	3·9(0·6)		3·9(0·3)		3·7(1·0)	

* Values are mean(s.d.). MPT, maximum phonation time; *f*0, fundamental frequency; HNR, harmonic to noise ratio; CPP, cepstral peak prominence; CPPS, smoothed cepstral peak prominence.

† ANOVA.

**Table 8:** Objective voice outcome measures for each time point, stratified for histopathology outcome

			After surgery*			
Pathology	Before surgery*	*P* †	1 month	*P* †	3 months	*P* †
MPT (s)		0·234		0·197		0·178
Benign	16·7(6·1)		14·9(6·4)		13·0(6·8)	
Malignant	18·8(10·1)		17·0(8·0)		15·4(8·4)	
*f*0 (Hz)		0·715		0·733		0·631
Benign	187·4(54·4)		193·8(51·9)		195·2(43·9)	
Malignant	182·5(59·9)		198·1(50·4)		189·9(38·8)	
*f*0 (lowest) (Hz)		0·096		0·328		0·738
Benign	129·9(32·2)		128·4(36·2)		128·6(31·8)	
Malignant	115·9(39·9)		119·0(44·8)		125·8(31·1)	
*f*0 (highest) (Hz)		0·067		0·944		0·842
Benign	527·5(205·8)		461·8(190·9)		512·0(217·6)	
Malignant	432·5(217·7)		465·2(209·3)		501·3(129·2)	
HNR (Hz)		0·243		0·614		0·283
Benign	20·5(4·1)		20·4(4·1)		20·8(3·8)	
Malignant	19·2(4·4)		19·9(4·1)		19·8(3·7)	
CPP (dB)		0·574		0·587		0·968
Benign	12·3(0·8)		12·2(0·8)		12·3(0·8)	
Malignant	12·2(0·8)		12·1(0·9)		12·3(0·8)	
CPPS (dB)		0·115		0·306		0·428
Benign	4·3(0·6)		4·2(0·6)		4·2(0·6)	
Malignant	4·0(0·6)		4·1(0·6)		4·2(0·6)	

* Values are mean(s.d.). MPT, maximum phonation time; *f*0, fundamental frequency; HNR, harmonic to noise ratio; CPP, cepstral peak prominence; CPPS, smoothed cepstral peak prominence.

† ANOVA.

## Discussion

IONM during thyroid surgery is widely used, frequently employing the standards set by the International Neural Monitoring Study Group^7^ ^16^. The present study has shown that IONM contributed to reliable identification of the RLN and EBSLN in 0·6 and 23·3 per cent of nerves respectively. The impact of reliable identification of the RLN and EBSLN with IONM correlated with clinical preservation of voice outcome after thyroid surgery.

In this cohort, transection of the strap muscles while preserving the ansa cervicalis was routine. The results showed that this element of the surgery did not impair voice outcome. Overall, there were no clinically relevant changes in voice‐related quality of life in the study group regarding patient‐reported outcomes, with a stable median VHI‐10 score of 2·0 before and after surgery, whereas the normal score is less than 7. Objective measurements performed by an independent laryngologist and speech–language therapist also showed no clinically relevant changes in all six variables after surgery.

The VHI‐10 score represents the patient's experience of voice changes. Of all clinical factors tested for their influence on VHI‐10 outcomes, none resulted in a prolonged reduction of voice outcome. In contrast to a previous report^17^ concluding that strap muscle division impairs voice outcome, the present results show that meticulous identification of all nerves is the main contributor to maintaining vocal outcome.

Subgroup analyses assessing the influence of lymph node dissection, total thyroidectomy, histopathological outcome and intraoperative loss of signal found no clinically significant changes in voice outcome, despite some reaching statistical significance. None of these, however, appeared to reflect clinically significant or long‐term impairment of vocal function. Nonetheless, CPP and CPPS were significantly lower after 3 months in patients who had loss of signal during operation. This was not the case at 1 month and could not be explained by a mutual reduction after 1 month in combination with lack of recovery for patients with loss of signal. This may be a late result of intraoperative loss of signal.

The main limitation of this study was the lack of a control group for direct comparison of the effect of nerve monitoring on voice outcome. The studied cohort was also small. The rare nature of nerve damage means that very large numbers of patients would be needed to generate sufficient power for the study. Cricothyroid electromyography was not performed in this study, although the rate of EBSLN injury was assessed by identification of cricothyroid twitch and indirectly during follow‐up by videostroboscopy.

Thyroidectomy with IONM confirmation of postdissection RLN and EBSLN function and routine division of strap muscles resulted in no clinically significant voice change as measured in an independent laryngology clinic.

## Disclosure

The authors declare no conflict of interest.

## Supporting information


**Appendix S1** Standardized voice recording/assessment protocol
