# Supplementary material for: Influence of neural monitoring during thyroid surgery on nerve integrity and postoperative vocal function
Source: BJS Open. 2018 Apr 25;2(3):135–41. doi: 10.1002/bjs5.50 (PMC5989980; doi:10.1002/bjs5.50)
Supplement: Supplementary file 1 — Appendix S1 Standardized voice recording/assessment protocol [file BJS5-2-135-s001.docx]

**BJS5_50**

**Influence of neural monitoring during thyroid surgery on nerve integrity and postoperative vocal function**

**A. F. Engelsman, S. Warhurst, S. Fraser, D. Novakovic and S. B. Sidhu**

**Appendix S1** Standardized voice recording/assessment protocol

**Tasks to for acoustic analysis:**

1. **Monologue in conversational speech (30 sec)**

- “Tell me, for about 30 seconds, what you have been up to in the last few days”

1. **Reading of the Rainbow Passage**

- “In your normal speaking voice, please read this passage.”

**The Rainbow Passage**

“When the sunlight strikes raindrops in the air, they act like a prism and form a rainbow. The rainbow is a division of white light into many beautiful colours. These take the shape of a long round arch, with its path high above, and its two ends apparently beyond the horizon. There is, according to legend, a boiling pot of gold at one end. People look, but no one ever finds it. When a man looks for something beyond his reach, his friends say he is looking for the pot of gold at the end of the rainbow”

1. **Reading of phrases**

- “Please read the following phrases” – CAPE-V and vowel onset phrases:

**Cape-V Phrases for Acoustic Analysis**

1. The blue spot is on the key again
2. How hard did he hit him?
3. We were away a year ago
4. We eat eggs every Easter
5. My mama makes lemon jam
6. Peter will keep at the peak

**Vowel onset Phrases**

1. Elliot ate an apple and allowed Andrew another
2. Each and every avenue is open at eight o’clock
3. Over on Ashton Avenue is an open air arena
4. I am in agreement in every aspect of our association
5. Alan’s attitude is overly obnoxious
6. In April, Adam always attends an extravaganza in Arizona
7. **MPT**

- “Again in your normal speaking voice say ‘ah’”, patient says a short ‘ah’
- “Now say it for a bit longer”, patient says a longer ‘ah’
- “Now take a big breath and hold that same ‘ah’ for as long as you possibly can
- After first attempt, “Great, now we’re going to do the same thing two more times”

1. **Counting**

- “Please count from 1 to 10”
- “Now please count from 80 to 90”

1. **Pitch Range**

- Mid-range to highest possible (3 attempts) – “On an /i/ sound, start at your normal speaking pitch and slide all the way to your highest note. You can stop at any time, take a breath and keep going”, *patient attempts,* “Great now do it again and try to go a bit higher” (last sentence x 2)
- Mid-range to lowest possible pitch (3 attempts) – “Again, on an /i/ sound, start at your normal speaking pitch and slide all the way down to your lowest note”, *patient attempts,*  “Great now do it again and try and slide even lower.”

1. **Singing**

- Please sing me “Happy Birthday”
